# Malignant Mesothelioma Mortality in Women — United States, 1999–2020

**DOI:** 10.15585/mmwr.mm7119a1

**Published:** 2022-05-13

**Authors:** Jacek M. Mazurek, David J. Blackley, David N. Weissman

**Affiliations:** 1 Respiratory Health Division, National Institute for Occupational Safety and Health, CDC.

Inhalation of asbestos fibers can cause malignant mesothelioma, a rapidly progressing and lethal cancer of the mesothelium, the thin layer of tissues surrounding internal organs in the chest and abdomen. Patients with malignant mesothelioma have a poor prognosis, with a median survival of 1 year from diagnosis. The estimated median interval from initial occupational asbestos exposure to death is 32 years (range = 13–70 years) (*1*). Occupational asbestos exposure is most often reported in men working in industries such as construction and manufacturing; however, women are also at risk for exposure to asbestos fibers, and limited data exist on longer-term trends in mesothelioma deaths among women. To characterize deaths associated with mesothelioma and temporal trends in mesothelioma mortality among women in the United States, CDC analyzed annual Multiple Cause of Death records from the National Vital Statistics System for 1999–2020, the most recent years for which complete data are available. The annual number of mesothelioma deaths among women increased significantly, from 489 in 1999 to 614 in 2020; however, the age-adjusted death rate per 1 million women declined significantly, from 4.83 in 1999 to 4.15 in 2020. The largest number of deaths was associated with the health care and social assistance industry (89; 15.7%) and homemaker occupation (129; 22.8%). Efforts to limit exposure to asbestos fibers, including among women, need to be maintained.

Malignant mesothelioma deaths were identified for 1999–2020 and included any death certificates for which an *International Classification of Diseases, Tenth Revision* (ICD-10) code for malignant mesothelioma was listed in the CDC WONDER Multiple Cause of Death mortality data.* Given the predominantly occupational etiology and long latency of mesothelioma, analysis was limited to deaths of women aged ≥25 years. The annual death rate (per 1 million women) was age-adjusted to the 2000 U.S. standard population. Age-adjusted death rates were mapped by state using geographic information system software (MapInfo Pro v2019.3; Precisely). Joinpoint Regression Program^†^ software (version 4.9.0.0.; National Cancer Institute) was used to evaluate time trends in deaths and log-transformed age-adjusted rates. Standard information about the usual industry and occupation^§^ was identified in the 2020 NCHS Mortality Multiple Cause of Death file for decedents in 46 states and New York City.^¶^ Occupations classified according to the four-digit 2012 U.S. Census Bureau coding system and the two-digit simple industry recode based on the 2012 North American Industry Classification System** were examined using SAS software (version 9.4; SAS Institute).

During 1999–2020, 12,227 (age-adjusted death rate: 4.59 per 1 million women) malignant mesothelioma deaths occurred among women aged ≥25 years in the United States; 11,093 (90.7%) occurred among women aged ≥55 years, 11,447 (93.6%) occurred among White women, and 11,561 (94.6%) among non-Hispanic women (Table 1); 11,499 (94.0%) had malignant mesothelioma listed as the underlying cause of death. Mesothelioma deaths were classified as mesothelioma of pleura (968; 7.9%), peritoneum (1,119; 9.2%), pericardium (35; 0.3%), other sites (1,385; 11.3%), and unspecified location (8,842; 72.3%). The annual number of malignant mesothelioma deaths increased 25%, from 489 in 1999 to 614 in 2020 (p<0.001), and the annual age-adjusted death rate declined from 4.83 per 1 million women in 1999 to 4.15 in 2020 (p = 0.038). During 1999–2020, the annualized state mesothelioma age-adjusted death rate exceeded 6.0 per 1 million women in seven states: Louisiana, Maine, Minnesota, Montana, Oregon, Washington, and Wisconsin (Figure).

**TABLE 1:** Number and rate of malignant mesothelioma deaths among women aged ≥25 years,* by selected characteristics and year — United States, 1999–2020

Characteristic	No. of deaths (%)	Death rate^†^ (95% CI)^§^
**Total**	12,227 (100)	4.59 (4.50–4.67)
**Age group, yrs** ^¶^		
25–34	71 (0.6)	0.16 (0.12–0.20)
35–44	282 (2.3)	0.60 (0.53–0.67)
45–54	781 (6.4)	1.66 (1.54–1.77)
55–64	1,857 (15.2)	4.68 (4.47–4.89)
65–74	3,203 (26.2)	11.69 (11.29–12.10)
75–84	4,018 (32.9)	23.17 (22.45–23.88)
≥85	2,015 (16.5)	25.10 (24.00–26.20)
**Race**		
White	11,447 (93.6)	5.03 (4.93–4.93)
Black	550 (4.5)	2.02 (1.85–2.19)
Asian or Pacific Islander	179 (1.5)	1.58 (1.34–1.82)
American Indian or Alaska Native	51 (0.4)	2.82 (2.07–3.75)
**Ethnicity**		
Hispanic or Latino	643 (5.3)	2.98 (2.75–3.22)
Non-Hispanic or Latino	11,561 (94.6)	4.69 (4.60–4.77)
Unknown	23 (0.2)	NA
Anatomic site**		
Pleura	968 (7.9)	0.35 (0.33–0.37)
Peritoneum	1,119 (9.2)	0.42 (0.39–0.44)
Pericardium	35 (0.3)	0 (—)
Other	1,385 (11.3)	0.52 (0.49–0.55)
Unspecified	8,842 (72.3)	3.29 (3.22–3.36)
**Year**		
1999	489 (4.0)	4.83 (4.40–5.26)
2000	487 (4.0)	4.77 (4.34–5.19)
2001	486 (4.0)	4.66 (4.24–5.07)
2002	444 (3.6)	4.17 (3.78–4.56)
2003	499 (4.1)	4.64 (4.23–5.05)
2004	516 (4.2)	4.77 (4.35–5.18)
2005	556 (4.5)	4.99 (4.58–5.41)
2006	503 (4.1)	4.49 (4.10–4.89)
2007	531 (4.3)	4.67 (4.27–5.07)
2008	557 (4.6)	4.79 (4.39–5.19)
2009	559 (4.6)	4.66 (4.27–5.05)
2010	562 (4.6)	4.72 (4.33–5.12)
2011	543 (4.4)	4.40 (4.03–4.78)
2012	615 (5.0)	4.92 (4.52–5.31)
2013	621 (5.1)	4.89 (4.50–5.27)
2014	610 (5.0)	4.67 (4.29–5.05)
2015	550 (4.5)	4.12 (3.77–4.47)
2016	569 (4.7)	4.16 (3.81–4.50)
2017	672 (5.5)	4.85 (4.47–5.22)
2018	603 (4.9)	4.20 (3.86–4.54)
2019	641 (5.2)	4.36 (4.02–4.70)
2020	614 (5.0)	4.15 (3.81–4.48)
p-value for trend	<0.001	0.038

**Source**: CDC WONDER Multiple Cause of Death data. https://wonder.cdc.gov/mcd.html

**Abbreviation**: NA = not applicable.

* *International Classification of Diseases,* *Tenth Revision* codes C45.0 (mesothelioma of pleura), C45.1 (mesothelioma of peritoneum), C45.2 (mesothelioma of pericardium), C45.7 (mesothelioma of other sites), or C45.9 (mesothelioma, unspecified).

^†^ Age-adjusted deaths per 1 million women using 2000 U.S. standard population.

^§^ https://wonder.cdc.gov/wonder/help/mcd.html#Confidence-Intervals

^¶^ Age-specific deaths per 1 million women using 2000 U.S. standard population.

** The sum of individual site death totals exceeds the total number of deaths for any site because some decedents have more than one site of mesothelioma listed on their death certificates.

**FIGURE:**
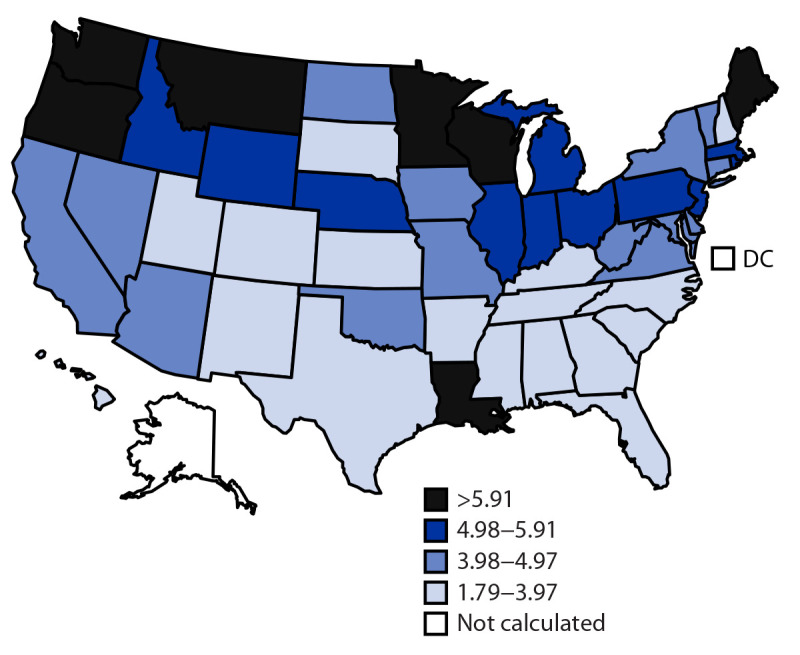
Malignant mesothelioma* annualized age-adjusted death rate^†^ per 1 million women aged ≥25 years — United States, 1999–2020 **Source**: CDC WONDER Multiple Cause of Death data. https://wonder.cdc.gov/mcd.html **Abbreviation**: DC = District of Columbia. * *International Classification of Diseases, Tenth Revision* codes C45.0 (mesothelioma of pleura), C45.1 (mesothelioma of peritoneum), C45.2 (mesothelioma of pericardium), C45.7 (mesothelioma of other sites), and C45.9 (mesothelioma, unspecified). ^†^ Adjusted using 2000 U.S. standard population. Age-adjusted death rates were not calculated for states with ≤20 malignant mesothelioma deaths (Alaska and DC).

Industry and occupation information was available for 567 (92.3%) of 614 malignant mesothelioma deaths among residents of 47 jurisdictions that occurred in women aged ≥25 years during 2020 (Table 2). Among 21 industry groups, the three with the most deaths were health care and social assistance (89; 15.7%); education services (64; 11.3%); and manufacturing (50; 8.8%). One hundred thirty-two occupations were reported on death certificates for malignant mesothelioma decedents among women during 2020. The three occupations with the highest numbers of mesothelioma deaths were homemakers (129; 22.8%); elementary and middle school teachers (32; 5.6%); and registered nurses (28; 4.9%).

**TABLE 2:** Usual industry and occupations* within industries associated with ≥5 malignant mesothelioma^†^ deaths among women aged ≥25 years — United States,^§^ 2020

Industry/Occupation	No. of deaths (%)
**Health care and social assistance**	89 (15.7)
Registered nurses	28 (4.9)
Nursing, psychiatric, and home health aides	11 (1.9)
Personal care aides	6 (1.1)
**Education service**	64 (11.3)
Elementary and middle school teachers	32 (5.6)
Teacher assistants	5 (0.9)
**Manufacturing**	50 (8.8)
Secretaries and administrative assistants	7 (1.2)
Production workers, all other	6 (1.1)
**Retail trade**	37 (6.5)
Retail salespersons	10 (1.8)
First-line supervisors of retail sales workers	9 (1.6)
Cashiers	5 (0.9)
**Public administration**	27 (4.8)
Secretaries and administrative assistants	6 (1.1)
**Finance and insurance**	24 (4.2)
**Professional, scientific, and technical services**	24 (4.2)
**Accommodation and food services**	23 (4.1)
Food service managers	5 (0.9)
Waiters and waitresses	5 (0.9)
**Other services (except public administration)**	23 (4.1)
Hairdressers, hairstylists, and cosmetologists	7 (1.2)
**Transportation and warehousing**	13 (2.3)
**Real estate and rental and leasing**	8 (1.4)
Real estate brokers and sales agents	6 (1.1)
**Administrative, support, and waste services**	8 (1.4)
**Information**	7 (1.2)
**Agriculture, forestry, fishing, and hunting**	6 (1.1)
**Construction**	6 (1.1)
**Arts, entertainment, and recreation**	5 (0.9)
**Other^¶^/Missing**	153 (27.0)
Homemaker	129 (22.8)

**Source**: NCHS Mortality Multiple Cause Files 2020. https://www.cdc.gov/nchs/data_access/vitalstatsonline.htm#Mortality_Multiple

**Abbreviation**: NCHS = National Center for Health Statistics.

* U.S. Census Bureau Industry and Occupation 2012 coding scheme. The industry grouping is the two-digit simple industry recode based on the 2012 North American Industry Classification System–informed codes obtained from the U.S. Census Bureau. Usual occupation is the occupation the person did for “most of his or her working life.” https://www.cdc.gov/nchs/data/dvs/Industry-and-Occupation-data-mortality-2020.pdf

^†^ *International Classification of Diseases, Tenth Revision* codes C45.0 (mesothelioma of pleura), C45.1 (mesothelioma of peritoneum), C45.2 (mesothelioma of pericardium), C45.7 (mesothelioma of other sites), or C45.9 (mesothelioma, unspecified). https://wonder.cdc.gov

^§^ Starting with the 2020 data year, NCHS and the National Institute for Occupational Safety and Health began a collaboration to translate industry and occupation information, submitted by 46 states and New York City (Arizona, North Carolina, Rhode Island, and District of Columbia did not participate in this program for 2020) to NCHS as part of their death certificate data, to U.S. Census Bureau Industry and Occupation codes (data collected from Iowa were inconsistent with other states’ data and were excluded). https://www.cdc.gov/nchs/data/dvs/Industry-and-Occupation-data-mortality-2020.pdf

^¶^ Includes mining, utilities, wholesale trade, and management of companies and enterprises.

## Discussion

Asbestos has been used in a variety of construction and manufacturing applications beginning in the 1930s. The annual use of asbestos in the United States peaked at 803,000 metric tons in 1973 and declined to approximately 320 metric tons in 2021 (*2*). Asbestos-related respiratory diseases and cancers are well recognized, and asbestos use is regulated by the Occupational Safety and Health Administration (*3*) and the Environmental Protection Agency (*4*). Despite the sharp decline in asbestos use, the findings in this report indicate that mesothelioma deaths among women continue to increase. Increases in total number, but not age-adjusted death rates, suggest that changes in underlying annual age distributions of the population over time are contributing to the observed increases in total mesothelioma deaths in women (*5*). Also, the observed increasing trend in the number of mesothelioma cases among women is consistent with a projection based on 1973–2005 Surveillance, Epidemiology, and End Results data (representing 10% of the U.S. population) that the number of mesothelioma cases among women would increase over time (*5*).

Among men, an estimated 85% of mesotheliomas were attributable to work-related asbestos exposure. Among women, the overall attributable risk was estimated at approximately 23% (*6*). Although occupational asbestos exposure is most often recognized among men working in shipbuilding, construction, manufacturing, and other industrial settings where women are less likely to be employed, exposure can also occur in other work settings as a consequence of disturbance of previously installed friable asbestos-containing materials during maintenance or renovation, or the resuspension of settled fibers in the air caused by dusting, sweeping, or cleaning (*7*). Exposures can also occur in work and nonwork settings through pathways, including potential environmental exposure to naturally occurring asbestos (*8*), indoors when older building materials containing asbestos are present, or from take-home exposures by indirect contact via family members who were exposed to asbestos fibers at workplaces outside of the home. In one study, the relative risk for mesothelioma among women with a husband or father working in an asbestos-related industry increased 10-fold (*9*). The geographic distribution of the highest mesothelioma death rates among women in states with a shipyard industry (e.g., Louisiana, Maine, Minnesota, Oregon, Washington, and Wisconsin) or past asbestos exposure associated with mining and processing vermiculite contaminated with asbestos (e.g., Montana) suggests that take-home asbestos exposure might affect disease development. Higher mesothelioma death rates in northern states might reflect greater use of asbestos in older building stock in that region.

The findings in this report are subject to at least six limitations. First, no information on exposure to asbestos or specific tasks performed at work are available on death certificates. Second, industry and occupation codes for deaths in 2020 were not compatible with coded information for previous years and resulted in a small number of observations in certain industries and occupations. Third, complete lists of all industries and occupations worked during life and information about family members’ work were not available. Fourth, the state issuing the death certificate might not always be the state in which decedent’s exposures occurred. Fifth, incomplete information on mesothelioma anatomic location on death certificates resulted in approximately 75% of all mesothelioma deaths classified as unspecified for anatomic location (i.e., ICD-10 code C45.9). Data from tumor registries indicate that approximately 74% of mesotheliomas among women arise from pleura (*10*). Using this proportion, approximately 9,050 mesothelioma of pleura deaths (411 per year) for 1999–2020 could be expected. Finally, mesothelioma cases with no histopathological evaluation might have been reported on death certificates as unspecified cancer and assigned less specific codes (e.g., ICD-10 code C76, malignant neoplasm of other and ill-defined sites) and therefore not captured in this analysis.

Efforts to limit exposure to asbestos fibers, including among women, need to be maintained. Although asbestos is no longer mined in the United States, as of early 2022 it is still imported and used (*2**,**4*). Moreover, in addition to contemporary cases arising from past exposures, cases associated with future occupational and environmental exposures might occur if activities such as remediation and demolition of older buildings and equipment are done with inadequate asbestos controls to protect workers and the surrounding community (*3*). Ensuring future decreases in mortality because of malignant mesothelioma will require meticulous control of exposures in activities such as ship and building renovation and demolition, and in asbestos remediation and disposal. Limiting exposure in workplaces outside of the home will help decrease take-home exposures and reduce family exposure. Clinicians should maintain a high index of suspicion for diseases caused by exposure to asbestos fibers when evaluating workers at risk for occupational exposure or their family members. The continuing risk for potential exposure to asbestos fibers underscores the need for ongoing surveillance to monitor temporal trends in malignant mesothelioma mortality; capturing information on industry and occupation for mortality data can help to provide meaningful interpretation of these trends.

**Summary**

**What is already known about this topic?**

Inhalation of asbestos fibers causes malignant mesothelioma. Although occupational asbestos exposure is most often recognized in men working in industries such as construction and manufacturing, women are also at risk for exposure.

**What is added by this report?**

The annual number of deaths with mesothelioma among women significantly increased, from 489 (age-adjusted death rate = 4.8 per 1 million women) in 1999 to 614 (4.2) in 2020. The largest number of deaths in 2020 was associated with the health care and social assistance industry (89; 15.7%) and homemaker occupation (129; 22.8%).

**What are the implications for public health practice?**

Efforts to limit exposure to asbestos fibers, including among women, need to be maintained.

## Footnotes

* ICD-10 codes C45.0 (mesothelioma of pleura), C45.1 (mesothelioma of peritoneum), C45.2 (mesothelioma of pericardium), C45.7 (mesothelioma of other sites), and C45.9 (mesothelioma, unspecified). https://wonder.cdc.gov/mcd.html

†
https://surveillance.cancer.gov/joinpoint/

§ Starting with the 2020 data year, CDC’s National Center for Health Statistics (NCHS) and the National Institute for Occupational Safety and Health (NIOSH) began a collaboration to translate industry and occupation information (submitted by 46 states and New York City [Arizona, North Carolina, Rhode Island, and District of Columbia did not participate in this program for 2020] to NCHS as part of their death certificate data) to U.S. Census Bureau Industry and Occupation codes. As part of this program, Census codes and industry and occupation groupings for 2020 usual occupation information was available and coded for 3,077,127 (91.7% of 3,354,879 records eligible for coding) decedents aged ≥15 years from 46 states and New York City (data collected from Iowa were inconsistent with other states’ data and were excluded). Usual occupation is the occupation the person did for “most of his or her working life.” https://www.cdc.gov/nchs/data/dvs/Industry-and-Occupation-data-mortality-2020.pdf

¶
https://www.cdc.gov/nchs/data_access/vitalstatsonline.htm#Mortality_Multiple

** The 2012 U.S. Census Bureau coding scheme was used to code industry and occupation narratives to individual four-digit census occupation and industry codes (https://www.census.gov/topics/employment/industry-occupation/guidance/code-lists.html) (referred to as 2010 Census Occupation Codes and 2012 Census Industry Codes). NCHS and NIOSH supplement the 2012 Census coding scheme with additional industry and occupation codes for decedents who were not in the civilian workforce, including “housewife/homemaker” which were also used in this report. The industry grouping is the two-digit simple industry recode based on the 2012 North American Industry Classification System–informed codes obtained from the U.S. Census Bureau.
